# Exploratory Analysis of Liraglutide Effects on Obstructive Sleep Apnea and Health‐Related Quality of Life in Individuals With Obesity and COPD: A Secondary Analysis of a Randomised Controlled Trial

**DOI:** 10.1111/cob.70079

**Published:** 2026-03-23

**Authors:** Sofie Krogh Wolsing, Ayse Dudu Altintas Dogan, Claus Bogh Juhl, Søren Hess, Torben Tranborg Jensen, Else‐Marie Bladbjerg, Ole Hilberg

**Affiliations:** ^1^ Department of Medicine University Hospital of Southern Denmark Vejle Denmark; ^2^ Department of Regional Health Research Faculty of Health Sciences, University of Southern Denmark Odense Denmark; ^3^ Department of Medicine Regional Hospital Horsens Horsens Denmark; ^4^ Department of Medicine University Hospital of Southern Denmark Esbjerg Denmark; ^5^ Steno Diabetes Center Odense Denmark; ^6^ Department of Nuclear Medicine Odense University Hospital Odense Denmark; ^7^ Unit for Thrombosis Research, Department of Clinical Biochemistry University Hospital of Southern Denmark Esbjerg Denmark

**Keywords:** COPD, GLP‐1 RA, HRQoL, obesity, OSA, overweight

## Abstract

Obstructive sleep apnea (OSA) is associated with chronic obstructive pulmonary disease (COPD) and obesity, and all three are linked to reduced health‐related quality of life (HRQoL). Coexistence of OSA and COPD increases morbidity and mortality compared to each condition alone. Liraglutide, a glucagon‐like peptide 1 receptor agonist, may influence respiratory and HRQoL outcomes. In this secondary exploratory analysis of a randomised controlled trial including individuals with overweight or obesity and COPD, we evaluated effects of liraglutide on OSA prevalence and severity, daytime sleepiness (Epworth Sleepiness Scale, ESS), and HRQoL (Short Form‐36 version 2, SF‐36v2). In a double‐blinded randomised controlled trial, 40 participants with overweight or obesity and COPD from two outpatient clinics were randomised to liraglutide (3.0 mg, subcutaneous) or placebo for 40 weeks. Cardiorespiratory monitoring, SF‐36v2 and ESS questionnaires were conducted at baseline and end of treatment. OSA was diagnosed in 84% of participants (70% in the liraglutide group and 85% in the placebo group). Compared to placebo, liraglutide significantly reduced OSA severity, with mean baseline‐adjusted differences of −9.87 events/h (β 95% CI −19.5; −0.247, *p* = 0.044) in Apnea–Hypopnea Index and −10.16 events/h (β 95% CI −19.29; −1.03, *p* = 0.029) in Oxygen Desaturation Index. ESS scores did not change significantly. Significant improvements were observed in the SF‐36v2 subdomains General Health Perception and Role Physical. OSA is common among individuals with overweight or obesity and COPD. Forty weeks of liraglutide treatment were associated with reduced OSA severity and improvements in selected HRQoL domains in this population.

## Highlights

- What Is Already Known About This Subject
  ○ In people with overweight or obesity and chronic obstructive pulmonary disease (COPD), obstructive sleep apnea (OSA) is a common comorbidity.
  ○ GLP‐1 RA (liraglutide) is effective for weight loss intervention in people with overweight or obesity and OSA.
- What this study adds
  ○ A weight loss intervention with GLP‐1 RA (liraglutide) reduces OSA severity and increases some domains of health‐related quality of life in people with overweight or obesity, OSA, and COPD.

**Abbreviations**
AHI: Apnea–Hypopnea Index
BMI: body mass index
COPD: chronic obstructive pulmonary disease
CPAP: continuous positive airway pressure
ESS: Epworth Sleepiness Scale
FEV1: forced expiratory volume in 1 s
FVC: forced vital capacity
GCP: good clinical practice
GLP‐1 RA: glucagon‐like peptide 1 receptor agonist
HRQoL: health‐related quality of life
ODI: oxygen desaturation index
OS: the overlap syndrome
OSA: obstructive sleep apnea
SF‐36v2: short form‐36 version 2

## 1 Introduction

Chronic Obstructive Pulmonary Disease (COPD) is characterised by irreversible airflow limitation and respiratory obstruction [1]. In 2019, COPD was the third most common cause of death worldwide, with 213.2 million prevalent cases reported globally [2]. The prevalence of obesity in people with COPD varies between 18%–54% [3]. The coexistence of COPD and obesity leads to worse dyspnea and poorer health‐related quality of life (HRQoL), lower functional capacity, and increased physical inactivity, which amplifies the need for health‐care utilisation [4].

Obstructive Sleep Apnea (OSA) is a breathing disorder characterised by narrowing of the upper airway, which impairs the normal ventilation during sleep and causes oxyhemoglobin desaturations and arousals leading to cognitive deterioration and disruption of sleep [5, 6]. OSA has been associated with decreased quality‐of‐life scores, as well as increased morbidity and mortality [7]. The most important risk factor for OSA is obesity due to fat accumulation in the neck tissues and constriction of the upper airway [8], and the vast majority of people with OSA are overweight or obese [6, 7]. The most common treatment for OSA is the continuous positive airway pressure (CPAP) treatment, which decreases the severity of OSA measured by the apnea‐hypopnea index (AHI), the oxygen desaturation index (ODI), and the daytime sleepiness, which can be measured by the Epworth Sleepiness Scale (ESS) [9].

The coexistence of OSA and COPD, called ‘the overlap syndrome’ (OS), increases the risk of mortality and morbidity compared to OSA and COPD alone [5]. Additionally, people with OS have reduced quality of life, more severe respiratory symptoms, a higher relative risk of hospitalisations and exacerbations than people with one disease state alone [10]. Therefore, investigating interventions on obesity to reduce the severity of OSA in people with COPD is highly relevant.

The effect of lifestyle and surgical induced weight loss as treatment for OSA has been investigated in several studies [11, 12, 13, 14]. Pharmacological weight loss interventions have shown positive effects in individuals with OSA; however, it remains uncertain whether these results apply directly to individuals with concurrent COPD. Patients with COPD are often excluded or underrepresented in OSA trials, and clinical characteristics including altered respiratory mechanics, nocturnal hypoxia, and systemic inflammation differ from those in typical OSA populations without COPD [15]. Therefore, results from populations without COPD may not be directly generalizable. To our knowledge, no previous studies have evaluated whether treatment with liraglutide, a Glucagon‐like peptide 1 receptor agonist (GLP‐1 RA), can reduce OSA severity in this specific population.

This study is a secondary exploratory analysis of a randomised controlled trial primarily designed to investigate the respiratory effects of liraglutide on pulmonary function in people with COPD and overweight or obesity [16]. In the present publication, we focus on whether liraglutide treatment, compared with placebo, leads to a significant reduction in the severity of obstructive sleep apnea, assessed by changes in the AHI and ODI. Furthermore, we evaluate whether treatment with liraglutide results in greater improvements in daytime sleepiness using the ESS, relative to placebo. Finally, this study examines whether changes in the Short‐Form Health Survey version 2 (SF‐36v2) domain scores differ significantly between the liraglutide and placebo groups, in order to assess HRQoL alterations in this population.

## 2 Materials and Methods

### 2.1 Study Design

This is an exploratory study of a 44‐week prospective, randomised, placebo‐controlled, double‐blinded, two‐centre, parallel‐group trial [16]. The study was conducted at the Department of Pulmonary Diseases, University Hospital of Southern Denmark (Esbjerg, Denmark), and the Department of Medicine, Section of Pulmonary Diseases, University Hospital of Southern Denmark (Vejle, Denmark).

Study procedures were in accordance with the Declaration of Helsinki after approval by the Scientific Ethics Committee of The Region of Southern Denmark (j. no. S‐20170147) and EudraCT (j. no. 2017–003551‐32). The study was registered at clinicaltrials.gov (NCT03466021) and monitored according to Good Clinical Practice (GCP) by the GCP Unit of Odense University Hospital (Odense, Denmark). Participants were continuously and systematically monitored and assessed for potential side effects throughout the study [16].

### 2.2 Study Participants

We recruited 40 participants from the outpatient clinics at the two participating centres and randomised the participants to treatment with either liraglutide (titration up till 3.0 mg, subcutaneous) or placebo in a 1:1 manner using an electronic device, with 20 participants in each group initially before dropouts. Both participants and clinical personnel were blinded to the randomisation code. If deemed necessary, the investigator could break the code.

Inclusion criteria were COPD, defined as forced expiratory volume in 1 s relative to forced vital capacity (FEV1/FVC) < 70% after maximal bronchodilation in accordance with the Global Initiative for Chronic Obstructive Lung Disease guidelines; former smoker; body mass index (BMI) > 27 kg/m^2^; and age 40–70 years.

Exclusion criteria were current smoker; treatment with systemic corticosteroids; diabetes mellitus of any type; interstitial pulmonary disease; severe hepatic, renal or heart disease; pancreatitis; pregnancy; or breastfeeding. All participants provided written informed consent.

### 2.3 Data Collection and Measurements

The first exploratory secondary outcome measure of the present study was to determine OSA prevalence and severity in the study population. Another exploratory secondary outcome measure was to investigate whether treatment with liraglutide, compared to placebo, resulted in a significant reduction in the severity of OSA, measured by changes in AHI and ODI for all participants. AHI, which is used to diagnose and assess the severity of OSA, implies the counted number of apneas (cessation of airflow for > 10 s) plus the number of hypopneas (various degrees of reduction in airflow resulting in desaturation) per hour during sleep [17]. AHI < 5 is normal, 5–14 is mild, 15–29 is moderate and > 30 is severe [18, 19]. ODI indicates the number of events that oxygen saturation decreases per hour, regardless of the duration [20]. ODI and AHI were determined based on cardiorespiratory data collection at baseline and at the end of the medication period (week 0 and 40, respectively) using the device Nox T3 portable monitor and Noxturnal v5.1 (Nox Medical, Reykjavík, Iceland; ResMed). One participant who was already treated with CPAP due to previous OSA diagnosis was asked not to use the device during the cardiorespiratory data collection. We instructed the participants to use the device for at least 4 h during sleep between 10 pm and 8 am on one night, preferably during the whole sleep period. If the device fell off early in the night, they were instructed to remount it. If no data were recorded, the process was repeated on a subsequent night.

An exploratory secondary outcome measure was to evaluate whether treatment with liraglutide compared to placebo resulted in greater improvements in daytime sleepiness using the ESS questionnaire. ESS measures sleepiness in the daytime, based on the respondents' rating (0–3) of the tendency to become sleepy in eight situations. Higher scores indicate severe symptoms [21, 22].

Another secondary exploratory outcome examined whether changes in SF‐36v2 domain scores differed significantly between the liraglutide and placebo groups in order to assess HRQoL alterations in this population. The SF‐36v2 measures eight health concepts: physical functioning, bodily pain, role limitations due to physical health problems, role limitations due to personal or emotional problems, emotional well‐being, social functioning, energy/fatigue, and general health perceptions. The results are often assessed in relation to norm‐based scores [23, 24, 25], which also were conducted in the present study.

Both ESS and SF‐36v2 are self‐administered surveys, which were performed at baseline and at the end of the medication period (0 and 40 weeks, respectively).

### 2.4 Statistical Analysis

Stata v18 (StataCorp LLC, TX, USA) was used for the statistical analysis. All variables were normally distributed. *p* < 0.05% was considered significant. A power calculation conducted for the primary publication [16] indicated the need for 32 participants. Accordingly, no post hoc power calculations were performed. Instead, treatment effects are presented with 95% confidence intervals to convey the magnitude and precision of the findings.

We evaluated the effect of liraglutide compared to placebo at the end of the treatment period (week 40) in terms of AHI, ODI, symptoms related to sleepiness using ESS, and HRQoL with SF‐36v2. The analyses included all participants with available cardiorespiratory monitoring data, which defines the population of interest for these outcomes. Mean baseline‐adjusted differences in AHI, ODI, ESS scores and SF‐36v2 (from baseline to the end of the medication period) in the liraglutide group compared to the placebo group were calculated by a linear mixed effects model and adjusted for age, gender, and body weight. Using linear mixed‐effects models allows inclusion of all available data and provides unbiased estimates under the assumption that missing data are missing at random. This approach accounts for missing measurements without requiring imputation. The analyses were conducted based on the intention‐to‐treat principle. Unblinding was performed after completion of the primary analyses. Blinding was maintained during the trial and the primary data analysis. The present exploratory secondary analyses were conducted after unblinding.

## 3 Results

### 3.1 Study Population

**FIGURE 1:**
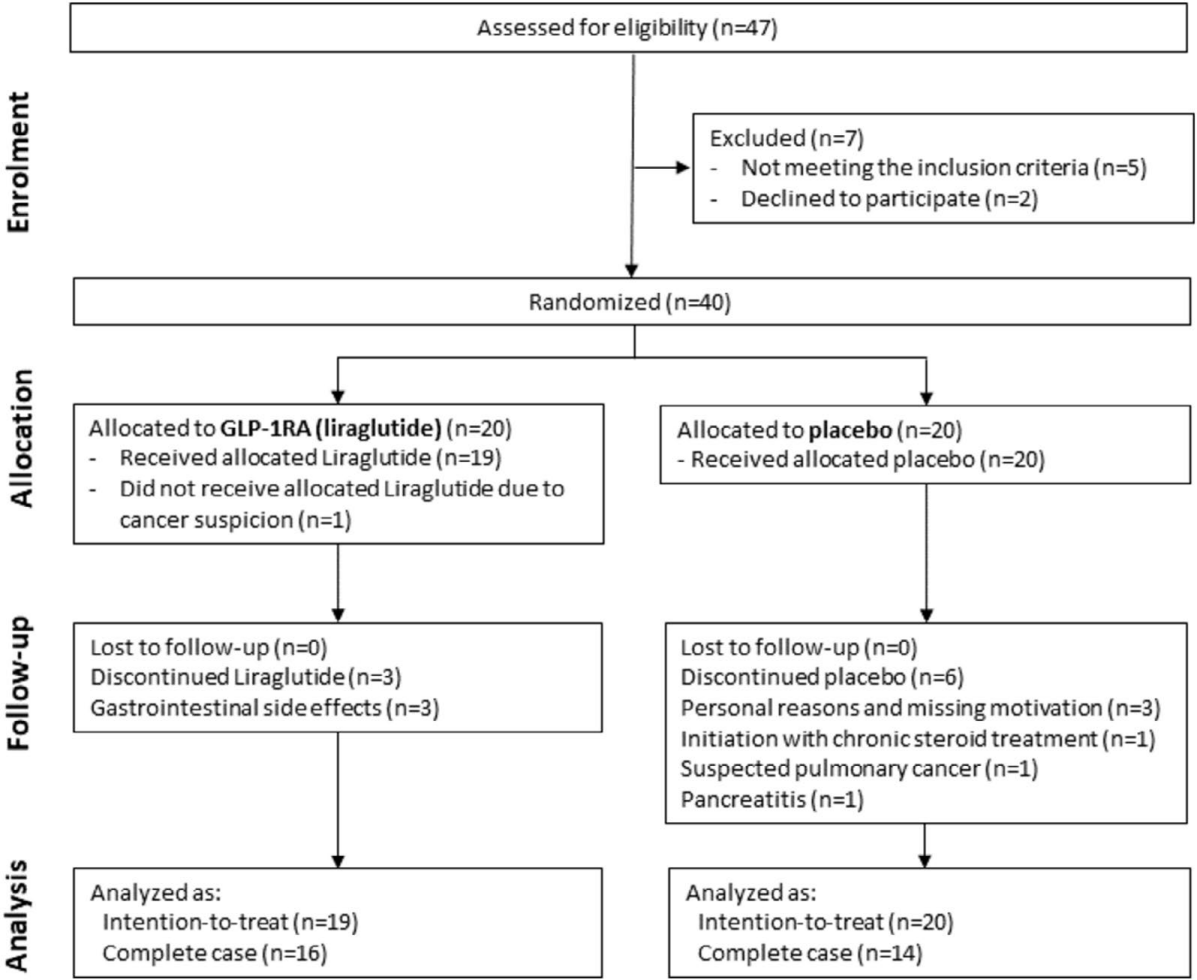
Flowchart of the trial. Flowchart from screening to data analyses in the liraglutide group and placebo group.

Of the 47 people assessed for eligibility, we randomised 40 participants for liraglutide or placebo treatment in a 1:1 manner between February 2018 and March 2020. The full set analysis consisted of 19 participants in the liraglutide group and 20 participants in the placebo group; cancer suspicion in one participant led to early discontinuation in the liraglutide group. The case complete analysis consisted of 16 participants in the liraglutide group and 14 participants in the placebo group (Figure 1).

### 3.2 Participant Characteristics

Table 1 presents baseline demographics, sleep parameters and HRQoL domains. A larger share of men participated than women, the mean age for all included was 64.7 ± 7.55 years and mean BMI was 35 ± 4.3. AHI and ODI were higher in the liraglutide group, but the differences were not statistically significant. FEV1 in percentage and FEV1/FVC ratio were significantly higher in the liraglutide group. Furthermore, there were differences in HRQoL domains, with a significantly higher score of the physical functioning domain in the placebo group.

**TABLE 1:** Baseline demographics, sleep parameters and health‐related quality of life domains^a^.

	Intention to treat		
	Liraglutide	Placebo	*p*
*n* (%)	20 (50.0)	20 (50.0)	
Sex (M/F)			
Female	7 (35.0)	9 (45.0)	
Male	13 (65.0)	11 (55.0)	0.52
Age	64.0 (8.4)	65.3 (6.7)	0.58
Height/cm	173.5 (7.9)	171.2 (9.2)	0.39
Weight/kg	103.9 (14.4)	104.0 (18.1)	0.98
BMI	34.5 (4.0)	35.4 (4.7)	0.52
OSA diagnosis	14 (70)	17 (85)	0.37
AHI‐index (events/h)	21.1 (13.9)	17.6 (12.2)	0.44
ODI‐index (events/h)	21.0 (14.3)	16.5 (10.4)	0.31
ESS total score	5.4 (3.2)	5.5 (2.5)	0.90
FEV1 best	1.83 (0.67)	1.41 (0.67)	0.06
FEV1 Pct	62.84 (17.84)	50.30 (20.20)	0.05
FVC best	3.16 (1.01)	2.83 (0.99)	0.32
FVC Pct	86.42 (19.11)	81.50 (19.34)	0.43
FEV1% FVC best	57.35 (9.59)	48.20 (11.91)	0.01
Physical component score	39.8 (5.7)	40.5 (5.7)	0.72
Mental component score	44.5 (8.4)	41.4 (9.8)	0.30
Physical functioning	47.7 (25.5)	66.3 (21.9)	0.02
Role physical	12.8 (10.9)	10.6 (10.9)	0.53
Bodily pain	72.0 (23.3)	60.5 (29.8)	0.19
General health perceptions	52.6 (24.3)	44.3 (24.8)	0.30
Vitality	55.3 (55.3)	49.7 (22.0)	0.39
Social functioning	80.9 (25.1)	76.3 (26.3)	0.57
Role emotional	18.0 (9.3)	14.5 (11.1)	0.30
Mental health	77.6 (15.7)	74.3 (16.7)	0.52

*Note:* Continuous variables are mean (SD). Categorical variables are *n* (%).

Abbreviations: AHI, apnea–hypopnea index; BMI, body mass index; ESS, Epworth Sleepiness Scale; FEV1, forced expiratory volume in 1 s; FVC, forced vital capacity; ODI, oxygen desaturation index; OSA, obstructive sleep apnea.

^a^ Baseline characteristics including anthropometric measures, pulmonary variables and 6‐min walking test have been published previously in reference [16]. Randomisation was performed for the primary COPD trial.

### 3.3 OSA Prevalence and Severity in the Study Population

**FIGURE 2:**
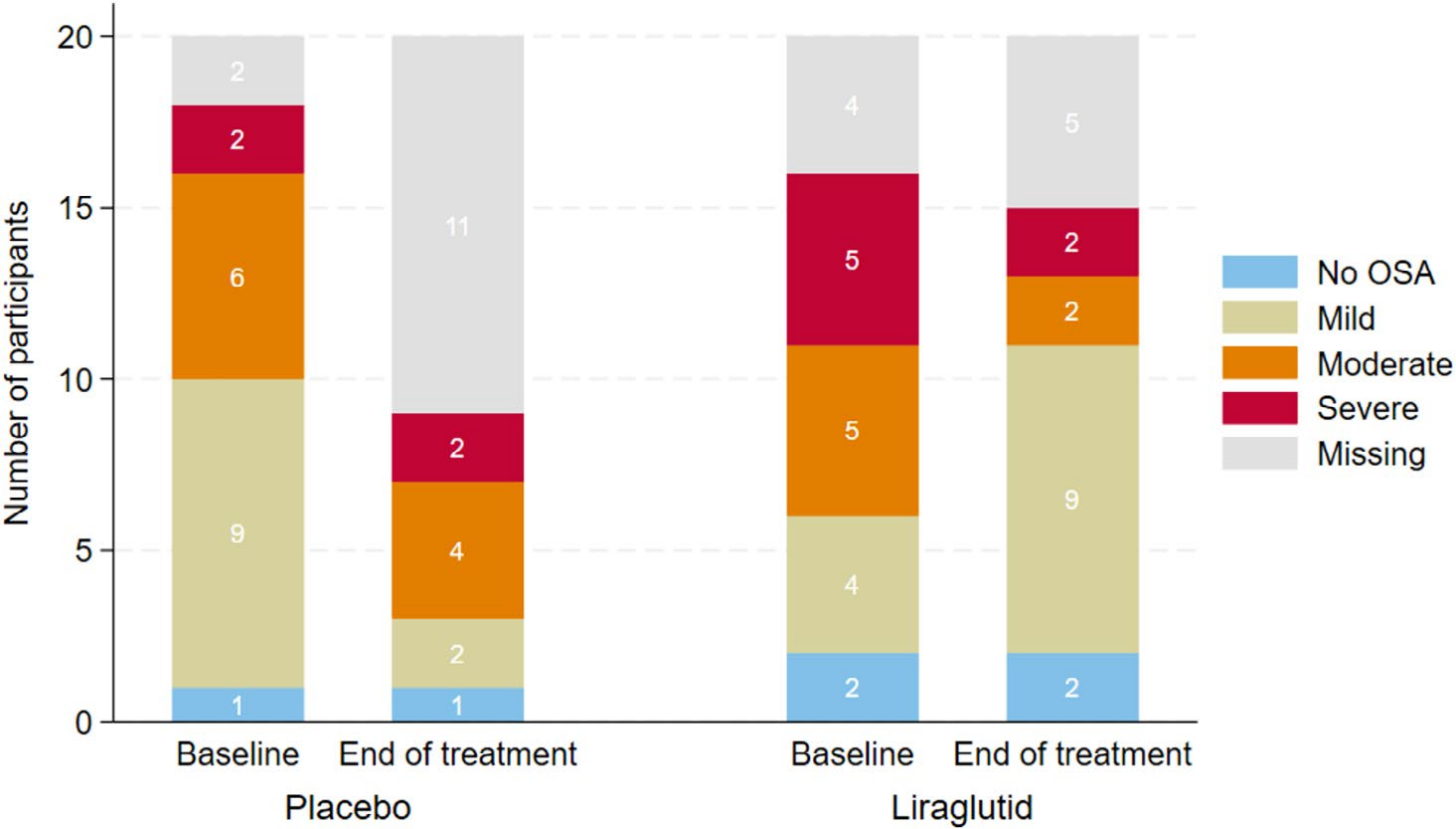
Prevalence and severity distribution of OSA in the study cohort, based on AHI classification criteria before and after end of treatment. *Missing involve both missing values for participants who completed the study and study dropouts.

We diagnosed 84% of the participants with some degree of OSA. Figure 2 illustrates the distribution of OSA prevalence and severity categories based on AHI classification criteria at baseline and at end of treatment for all participants. Visually, a higher proportion of participants in the liraglutide group appeared to be classified with mild OSA at end of treatment compared with baseline, whereas this pattern was less apparent in the placebo group. However, the figure is descriptive and does not account for individual transitions between OSA severity categories. Missing data, including both dropouts and missing measurements among study completers, limit a direct comparison between groups.

### 3.4 Effect of Treatment With Liraglutide on OSA

Table 2 presents AHI, ODI and ESS scores. There was a significant change of −10.59 events per hour (β 95% CI −20.19;−0–98, *p* = 0.031) in the crude analysis and −9.87 events per hour (β 95% CI −19.5;−0.247, *p* = 0.044) in the mean baseline adjusted difference of AHI. In ODI, the crude change was −10.55 events per hour (β 95% CI −19.68; −1.41, *p* = 0.024) and the mean baseline adjusted difference was −10.16 events per hour (β 95% CI −19.29; −1.03, *p* = 0.029). Both these analysis show that liraglutide significantly decreased OSA severity. No significant decrease in ESS score were found. The score decreased by 0.33 points (β 95% CI −2.53;1.87, *p* = 0.768) in the crude analysis, and when adjusted for mean baseline differences, the decrease was 0.42 points (β 95% CI −2.6; 1.77, *p* = 0.708).

**TABLE 2:** Crude and mean baseline adjusted differences in AHI, ODI and ESS from baseline to the end of treatment period in the liraglutide group compared to the placebo group.

	AHI at baseline (mean ± SD)	AHI at follow‐up (mean ± SD)	Crude between‐group difference (β [95% CI])	*p*	Adjusted between‐group difference (β [95% CI])	*p*
Placebo	17.58 ± 12.16	19.41 ± 15.11				
Liraglutide	21.06 ± 13.93	13.35 ± 12.12	−10.59 [−20.19;−0.98]	0.031	−9.87 [−19.5;−0.247]	0.044

	ODI at baseline (mean ± SD)	ODI at follow‐up (mean ± SD)	Crude between‐group difference (β [95% CI])	*p*	Adjusted between‐group difference (β [95% CI])	*p*
Placebo	16.51 ± 10.43	19.88 ± 15.76				
Liraglutide	21.04 ± 14.33	14.86 ± 13.31	−10.55 [−19.68;‐1.41]	0.024	−10.16 [−19.29;‐1.03]	0.029

	ESS at baseline (mean ± SD)	ESS at follow‐up (mean ± SD)	Crude between‐group difference (β [95% CI])	*p*	Adjusted between‐group difference (β [95% CI])	*p*
Placebo	5.5 ± 2.48	4.73 ± 2.53				
Liraglutide	5.38 ± 3.2	4.46 ± 2.5	−0.33 [−2.53;1.87]	0.768	−0.42 [−2.6;1.77]	0.708

*Note:* Numbers are mean ± SD. AHI and ODI are in events per hour.

Abbreviations: AHI, apnea–hypopnea index; ESS, Epworth Sleepiness Scale; ODI, oxygen desaturation index.

### 3.5 Effects of Treatment With Liraglutide on Health‐Related Quality of Life

Table 3 presents average baseline‐adjusted group differences and norm‐based differences for domains and subdomains of SF‐36v2 (Table 3). There were significant average baseline‐adjusted group differences and norm‐based differences for the liraglutide group compared to placebo, for the subdomain Role Physical. This shows that participants experienced fewer limitations in daily activities in relation to physical health [26]. Furthermore, a significant difference was found in the General Health Perceptions subdomain, which demonstrates that participants achieved improved perceptions of overall health and future health [26]. Significant differences were not observed in the other domains, neither in relation to norm‐based scores. However, the subdomains Physical Functioning and Vitality had near‐significant differences. These describe the ability to conduct physical activities and measure energy levels and fatigue, respectively.

**TABLE 3:** Average baseline‐adjusted group differences (liraglutide—placebo) and norm‐based differences for domains and subdomains of SF‐36v2.

Domains	ΔEOT between groups	*p*	NBS	*p*
Physical component score	0.51	0.76		
Mental component score	4.43	0.11		
Physical functioning	−11.2	0.09	−4.3	0.094
Role physical	9.13	0.004	3.3	0.004
Bodily pain	−0.9	0.89	−0.4	0.89
General health perceptions	13.1	0.045	6.2	0.045
Vitality	11.3	0.068	5.4	0.068
Social functioning	10.6	0.185	4.3	0.185
Role emotional	3.1	0.317	1.3	0.317
Mental health	3.7	0.47	2.1	0.47

Abbreviations: EOT, end of treatment; NBS, norm‐based score.

## 4 Discussion

To our knowledge, this is the first study to evaluate the effect of liraglutide treatment on OSA in people with overweight or obesity and COPD. We examined the effect of liraglutide (3.0 mg) for 40 weeks on measures related to OSA and HRQoL.

We found that treatment with liraglutide in 40 weeks resulted in significant decreases in AHI‐ and ODI events per hour, but not ESS score. Furthermore, treatment led to improvements in participants’ perception of general health and role physical, in accordance to the SF‐36v2 questionnaire.

Previous studies have established that weight loss intervention with liraglutide reduces OSA severity [27, 28, 29]. However, no studies have tested this in people with overlap syndrome. A meta‐analysis published by Li et al. [30] in 2025 involving 1032 participants in six different studies compared changes in AHI for participants with OSA who received treatment with the GLP‐1RA tirzepatide or liraglutide, in relation to placebo. The analysis showed an accumulated significant decrease of 9.48 events per hour (95% CI = −12.56 to −6.40, *I* ^2^ = 92%) in favour of active treatment with GLP‐1RA. This is in line with our result of −10.59 events per hour (β 95% CI −20.19;−0–98, *p* = 0.031) in the crude analysis and −9.87 events per hour (β 95% CI −19.5;−0.247, *p* = 0.044) in the mean baseline adjusted difference of AHI. The meta‐analysis showed that the GLP‐1 RA tirzepatide significantly reduced AHI more than liraglutide. However, liraglutide had a more pronounced reduction in the severity of OSA in lower doses [30]. In our study some participants experienced gastrointestinal side effects to the medication [16], which are consistent with the known side effect profile of GLP‐1 RAs. This is a limitation to treatment with GLP‐1 RA, as it might affect adherence and treatment satisfaction [31].

In our study, participants diagnosed with OSA were offered to start CPAP treatment. Out of 22 participants diagnosed with moderate to severe OSA, 15 accepted treatment. It was expected that daytime sleepiness would be improved for the study participants treated with both CPAP and study medication. However, in an additional explorative data analysis, we were not able to detect a significant difference in ESS score from baseline to end of treatment between the liraglutide and placebo groups when only including participants using CPAP. The SCALE study by Blackman et al. [27] had the same conclusion with no significant difference in ESS between liraglutide and placebo groups in an OSA population, but a post hoc analysis revealed that a greater weight loss was associated with a greater decrease in ESS score. In addition, a study involving people with OSA who underwent bariatric surgery to induce weight loss also did not observe a significant change in ESS scores [32]. However, daytime sleepiness might not be universal for people with OSA, and notably, ESS might be inaccurate in predicting OSA in people with COPD, as these persons may experience daytime fatigue instead in relation to COPD symptoms [8].

In people with COPD, obesity is common, and the number of comorbidities increases with the severity of obesity. This combination is associated with worse COPD‐related outcomes such as impaired quality of life [33]. In a cohort study with different severities of OSA, the authors found that severe OSA, higher age and higher BMI were associated with lower SF‐36v2 physical component summary measure score [34]. We found significant improvements in two of the eight health domains of SF‐36v2: General Health Perceptions and Role Physical. Although these changes were significant with a 13.1 point improvement in General Health Perceptions and a 9.1 point improvement in Role Physical, it is at the border of clinical importance. Studies in people with COPD have suggested clinically important differences are 15 points for General Health Perceptions and 12.5 points for Role Physical [35]. However, some individuals might still experience clinically relevant improvement. Our results are in line with the abovementioned study by Blackman et al. [36] who also found greater improvement of the domain General Health Perceptions from the SF‐36v2 questionnaire.

COPD and obesity are inflammatory diseases, and both predispose individuals to OSA. Intermittent hypoxemia is comparable to ischemia–reperfusion injury, leading to oxidative stress and contributing to reactive oxygen species and inflammatory mediator production, which triggers upper airway and systemic inflammation [20]. It is likely that the presence of OSA affects HRQoL in this overweight and obese population with COPD, and any accompanying treatment will improve HRQoL in this group. Thus, other studies underline the importance of OSA treatment for achieving a positive impact on HRQoL and patient well‐being [36].

The gold standard treatment for OSA is CPAP. However, compliance to CPAP is often an issue, which emphasises the need for alternative or additional treatment options [37]. Furthermore, CPAP does not in itself lead to weight loss [38, 39]. Though treatment of OSA with CPAP is the first and most important choice of treatment [40], it should also address underlying causes such as overweight and obesity [41, 42]. This knowledge and our study results rationalise additional treatment with liraglutide for people with OSA.

The inclusion criteria of our study were BMI > 27. Even though non‐obese people are at lower risk of OSA [43], studies show that liraglutide can reduce severity of OSA independent of weight loss [30]. This thesis originates from preclinical studies, which suggests that GLP‐1AR activation might increase respiratory drive and calm breathing rhythms [44].

### 4.1 Strengths and Limitations

The strengths of our study are the randomised controlled design, which is the gold standard for intervention studies [45]. The 40‐week follow‐up provided valuable insights into both objective sleep outcomes and subjective patient‐reported outcomes. Importantly, the study explores weight loss pharmacotherapy as an adjunct or alternative to CPAP, which offers a new perspective for OSA treatment in people with COPD.

However, several limitations should be acknowledged. We included and randomised 40 participants, but ended up with 30 completing participants due to comorbidities diagnosed during the study, invalid cardiorespiratory data, and dropouts. The sample size of this study, which was determined based on the power calculation for the primary outcome of the trial, represents an important limitation of the present exploratory analyses. The study was not specifically powered to detect changes in secondary outcomes such as AHI, ODI, daytime sleepiness (ESS) and HRQoL domains (SF‐36V2), which may have reduced the ability to detect statistically significant effects. To address missing data and participant dropouts, linear mixed‐effect models were applied, allowing inclusion of all available observations while accounting for within‐subject variability over time, thereby reducing bias associated with missing observations [46]. Nonetheless, the findings should be interpreted as exploratory, and confirmation in adequately powered prospective studies is warranted.

The baseline differences in lung function between the two groups might have influenced the AHI and ODI outcomes. Previous research has demonstrated that lower FEV_1_/FVC is significantly associated with higher AHI and ODI, while lower FEV_1_ is primarily associated with higher ODI [47, 48]. Therefore, this potential confounding factor should be taken into account when interpreting the observed OSA outcomes. A limitation of our analysis is that participants without baseline OSA were included in the linear mixed‐effects models, which may have diluted the observed effect on AHI and ODI. A subset analysis including only participants with baseline OSA was not feasible due to the small and unbalanced number of participants at the end of treatment.

Some participants were diagnosed with OSA, but either declined OSA treatment with CPAP or discontinued treatment due to side effects. This might confound the result of the ESS score, as CPAP treatment would be a mediating factor. For a more accurate result, participants should either not have received CPAP treatment at all or all should have received treatment.

Although the baseline prevalence of OSA was slightly different between groups, this difference was not statistically significant. As this secondary outcome was defined as change in AHI from baseline to end of treatment, baseline values are inherently accounted for in the change‐score analysis. However, small sample sizes may still make results vulnerable to baseline imbalances, and this should be considered when interpreting the findings. Finally, the modest sample size limits the ability to conduct meaningful subgroup analyses and reduces the generalizability of the findings.

### 4.2 Implications for Future Research and Clinical Practice

Future research should involve larger‐scale studies to validate and expand upon these findings.

Future clinical practice might implicate prioritising the systematic assessment and diagnosis of comorbid conditions such as OSA, which is associated with overweight and obesity in people with COPD. Furthermore, treatment strategies for OSA should extend beyond CPAP alone and consider weight loss interventions, including pharmacological options such as liraglutide.

## 5 Conclusion

In people with overweight or obesity and COPD, OSA is a common comorbidity. Forty weeks of treatment with the GLP‐1 RA liraglutide reduced AHI and ODI. ESS score at end of treatment was not improved, and only the General Health Perceptions and Role Physical subdomains were significantly improved measures from the SF‐36v2 questionnaire. Our study suggests that liraglutide may be an appropriate additional treatment option in people with overweight or obesity, COPD and OSA.

## Funding

This work was supported by the Novo Nordisk, the Karola Jørgensen Research Foundation, the Research Council of Hospital South West Jutland, University hospital of Southern Denmark and the Region of Southern Denmark.

## Conflicts of Interest

Claus Bogh Juhl serves as a speaker for Novo Nordisk but has no financial interest in the current study. The remaining authors declare no conflicts of interest.

## Data Availability

The data that support the findings of this study are available from the corresponding author upon reasonable request.
